# Physiological and Biochemical Changes Reveal Differential Patterns of Docosahexaenoic Acid Partitioning in Two Marine Algal Strains of *Isochrysis*

**DOI:** 10.3390/md15110357

**Published:** 2017-11-12

**Authors:** Zheng Sun, Yong Chen, Xuemei Mao, Jin Liu

**Affiliations:** 1 Key Laboratory of Exploration and Utilization of Aquatic Genetic Resources, Ministry of Education, Shanghai Ocean University, Shanghai 201306, China; zsun@shou.edu.cn (Z.S.); m150110190@st.shou.edu.cn (Y.C.); 2 International Research Center for Marine Biosciences, Ministry of Science and Technology, Shanghai Ocean University, Shanghai 201306, China; 3 National Demonstration Center for Experimental Fisheries Science Education, Shanghai Ocean University, Shanghai 201306, China; 4 Institute for Food and Bioresource Engineering and Department of Energy and Resources Engineering, College of Engineering, Peking University, Beijing 100871, China; mxuemei@pku.edu.cn

**Keywords:** Nitrogen deficiency, neutral lipids, DHA distribution, fatty acid profile

## Abstract

The marine microalgae *Isochrysis* are a good producer of natural docosahexaenoic acid (DHA). To better understand the patterns of DHA accumulation and distribution, two *Isochrysis* strains, CL153180 and CCMP462, were evaluated in this study. In a batch culture, CL153180 showed a decline in DHA content while CCMP462 exhibited a progressive increase during the late growth period when nitrogen was almost exhausted. In response to nitrogen deficiency (ND), both strains showed a considerable increase in neutral lipids (NL) at the expense of glycolipids (GL) but had little variation in phospholipids (PL). In CL153180, the DHA percentage of NL decreased gradually upon ND, while that in CCMP462 increased progressively to 21.4% after 4 days of ND, which is around 5-fold higher than CL153180. Accordingly, in contrast to CL153180 that stored DHA predominantly in GL, CCMP462 accumulated DHA mainly in NL in late days of ND. Taken together, we proposed a working model for the differential DHA partitioning patterns between two *Isochrysis* strains: for CCMP462, the degradation of GL released free fatty acids including DHA, which was incorporated into NL upon ND; whereas for CL153180, the released DHA from GL might not be incorporated into NL, and, consequently, might be subject to β-oxidation for degradation.

## 1. Introduction

As an omega-3 long-chain polyunsaturated fatty acid, docosahexaenoic acid (DHA), represents an essential substance for human metabolism and provides important physiological regulatory functions. DHA is a vital component of the brain cell membrane and retina, directly involved in the formation and development of brain cells for thinking and consolidating memory in fetuses and infants [1]. The consumption of supplementary DHA influences infant behavior and reduces the likelihood of developing allergies and colds [2]. DHA also improves visual acuity [3] and promotes the development of visual functioning [4]. In addition, it is anti-inflammatory, enhances immunity, prevents cardiovascular and cerebrovascular diseases (as well as cancer), and is also anti-hypertension [5].

Cold-water oceanic fish oils are rich in DHA and are currently the main source of DHA for human use. Fish is unable to de novo synthesize DHA but obtains it via bioaccumulation in the food chain. However, fish oil-derived DHA has its intrinsic disadvantages, such as declining supplies, an unpleasant odor, and difficulties in controlling the quality of fish oils [6], which drives the exploration of alternative sources for more sustainable DHA production. Marine microalgae, the primary producers of DHA in the aquatic food chain, are promising alternatives to fish oils and have received more and more attention from both industrial and academic communities. There have been a number of algae intensively studied with respect to their DHA producing capability, such as *Schizochytrium mangrovei* [7], *Schizochytrium limacinum* [8], *Aurantiochytrium* sp. [9], *Crypthecodinium cohnii* [10,11,12] and *Isochrysis* sp. [13]. Among them, *Isochrysis* sp. are regarded as a good candidate. In addition to the high DHA content, they also possess the following advantages: (1) fast growth rates; (2) easy extraction due to the lack of a cell wall; and (3) higher levels of antioxidants, which helps to avoid oxidative decomposition during the extraction process [14,15]. 

Liu et al. [13] conducted a comprehensive study of 19 natural *Isochrysis* strains from public algae collection centers, but lacked the time course comparison with respect to DHA accumulation and partitioning in different lipid classes. In this study, two *Isochrysis* strains, namely, CCMP462 and CL153180, were comparatively examined in a time course manner under both nitrogen-replete and nitrogen-depleted conditions. The results indicated the important role of nitrogen availability in affecting DHA synthesis and revealed distinct patterns of DHA accumulation and partitioning into neutral lipids between these two algal strains. Our data provide insights into future engineering of *Isochrysis* for improved production of DHA.

## 2. Results and Discussion

### 2.1. Biomass and Lipid Accumulation of Two Microalgae

**Figure 1:**
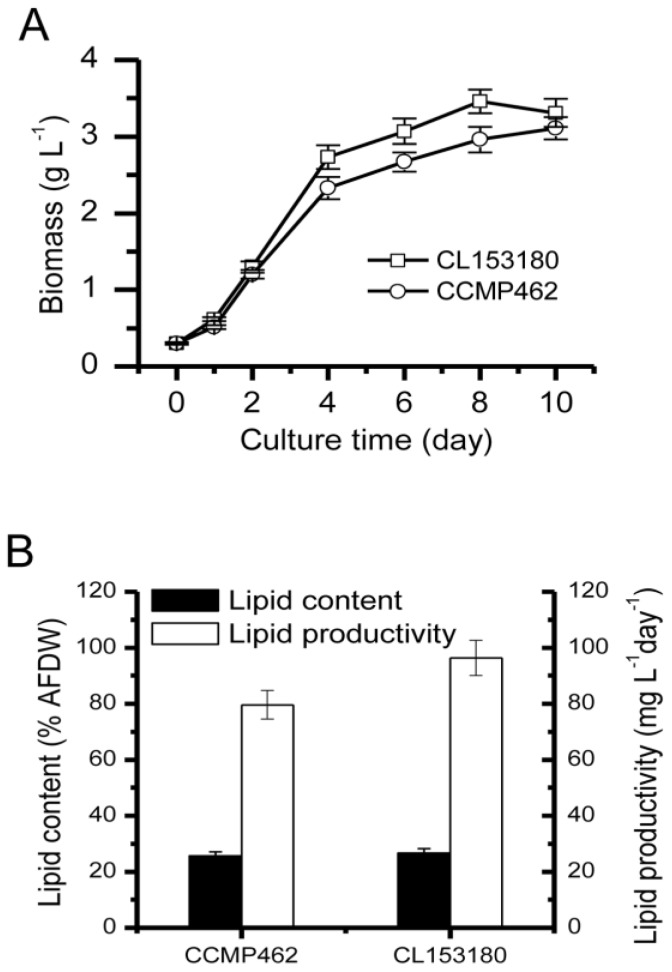
(**A**) Biomass accumulation; and (**B**) lipid production of CL153180 and CCMP462. Data were obtained from cells grown for 10 days.

As indicated in Figure 1A, the initial inoculation quantities for CCMP462 and CL153180 were both 0.3 g·L^−1^. After 10 day of culturing (continuous illumination: 100 µmol·m^−2^·s^−1^), the biomass reached over 3 g·L^−1^, which was higher than previous reports and may be explained by the difference in illumination. Cai et al. [16] reported that after 7 days of culturing *Isochrysis galbana* 8701 with continuous illumination at an irradiance of 72 µmol m^−2^·s^−1^, the biomass concentration reached 1.17 g·L^−1^. Yoshioka et al. [17] observed that under an irradiance of 20 µmol photons m^−2^·s^−1^, the biomass concentration of *Isochrysis galbana* after 6 day of culture increased to 1.09 g·L^−1^. According to a report by Liu et al. [13], biomass accumulation of *Isochrysis galbana* is proportional to illumination in the irradiance range of 30–120 µmol m^−2^·s^−1^.

Figure 1B indicates the lipid accumulation in CCMP462 and CL153180. The overall lipid contents of two *Isochrysis* strains were quite similar, both reaching up to 25–26% of the dry algal cell weight. According to previous reports, the overall lipid contents of *Isochrysis* generally account for 18–34% of the dry weight [18,19,20]. As for lipid productivity, the yield of CL153180 was relatively higher, reaching 96.29 mg·L^−1^·day^−1^ within 10 day of culturing, while the lipid productivity of CCMP462 was 79.61 mg·L^−1^·day^−1^.

### 2.2. Batch Culture for DHA Production

**Figure 2:**
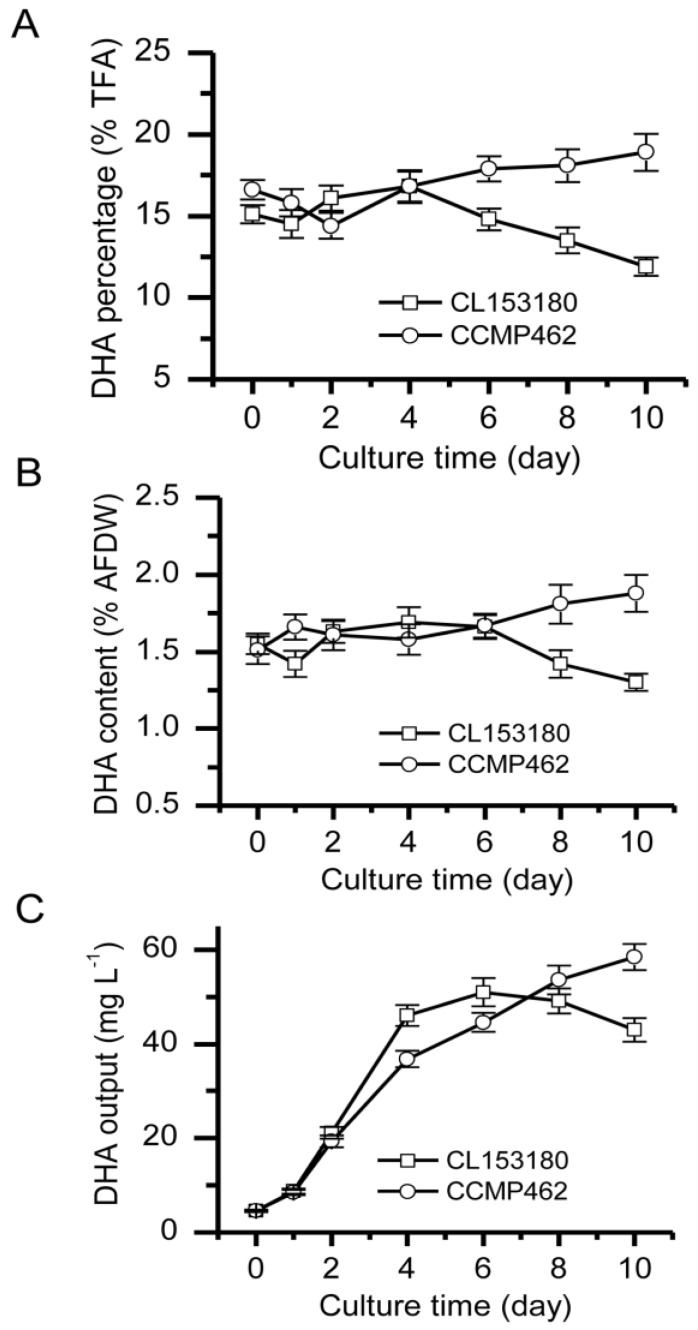
Time course of (**A**) docosahexaenoic acid (DHA) percentage; (**B**) DHA content; and (**C**) DHA output of CL153180 and CCMP462 in batch cultures.

The DHA accumulation in CCMP462 and CL153180 was further examined. Results demonstrated that the DHA content in CCMP462 increased continuously throughout the culturing period, while the opposite was observed in CL153180, whereby a change from an increasing trend to a decreasing trend was observed during the later stage of culturing. Specifically, the DHA percentage of CCMP462 in the overall lipids increased from 16.6 to 18%, while the maximum DHA percentage of CL153180 (16.8%) was reached on day 4, followed by a decrease to 11.9% from day 4 to day 10 (Figure 2A). Calculated with the ratio of DHA to the dry weight of the algal cells, the DHA content in CCMP462 gradually increased from 1.51 to 1.88%, while the DHA content in CL153180 initially increased to 1.69% (day 4) and then gradually decreased to 1.3% (Figure 2B). A similar trend was also observed with respect to DHA output: the DHA output of CCMP462 increased from 4.53 mg·L^−1^ to 58.46 mg·L^−1^, while for CL153180 the DHA output started decreasing to 43.03 mg·L^−1^ from day 6 (Figure 2C). It is evident that although CCMP462 and CL153180 both belong to *Isochrysis*, they feature distinct DHA accumulation patterns. From a DHA batch production point of view, CCMP462 is superior to CL153180 and may be more suitable for large-scale production. 

In a batch culture, the extension in incubation time will gradually deplete nutrients in the culture medium. Of all the nutrients, nitrogen is the most important one, closely associated with the lipid synthesis in microalgae. According to a report by Liu et al. [13], an initial nitrogen level for *Isochrysis* of about 100 mg·L^−1^ would be exhausted within 4–6 day of culturing. In the present study, a similar scenario was observed: the initial nitrogen content in the F/2 culture medium was 120 mg·L^−1^ and was nearly depleted within 4–6 day of culturing, which was closely associated with the turning point in DHA accumulation between the two strains. Therefore, we proposed that nitrogen deficiency (ND) stress may be an external factor inducing the different DHA accumulation patterns between CCMP462 and CL153180. Further experiments under ND conditions were conducted to verify this hypothesis.

### 2.3. Impact of ND on Lipid Compositions and Cell Structures

**Figure 3:**
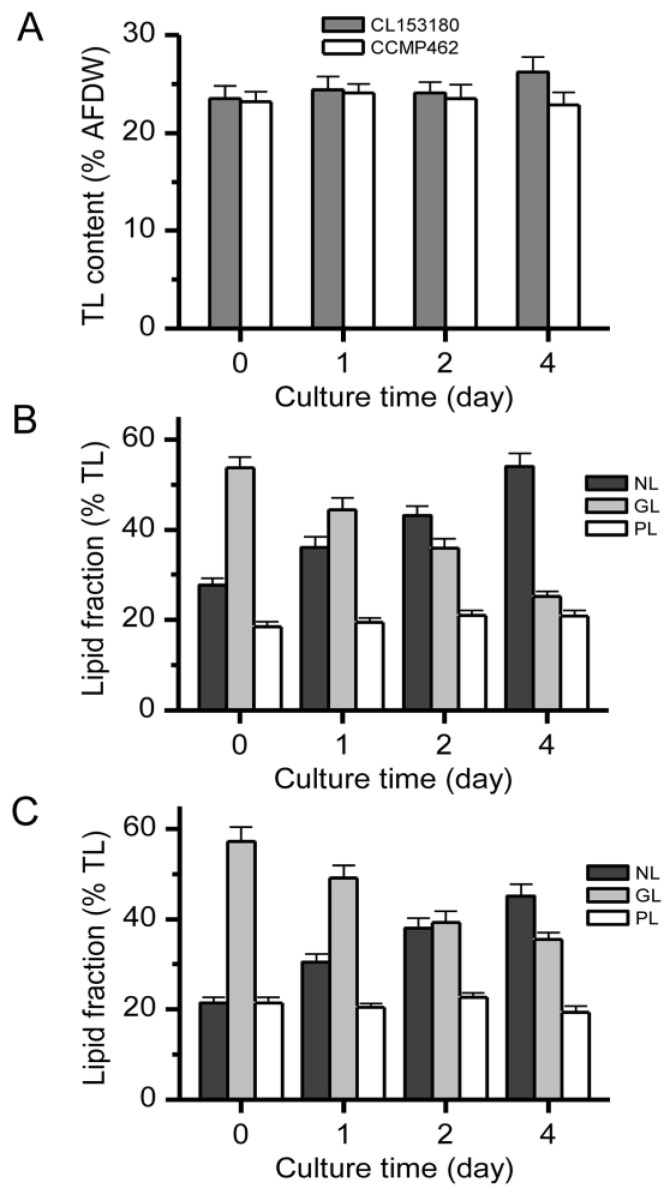
(**A**) Total lipid contents; (**B**) lipid fraction percentages of CL153180; and (**C**) lipid fraction percentages of CCMP462 under nitrogen-depleted growth conditions with continuous light illumination of 100 μmol photo m^−2^ s^−1^.

Under a continuous light illumination of 100 µmol photo m^−2^·s^−1^, CL153180 and CCMP462 were cultured for 4 day under ND conditions. The two microalgae possessed similar overall lipid contents at the beginning of the experiment. The lipid content of CL153180 increased slightly from 23.5% to 26.2% within 4 day (Figure 3A). The content of neutral lipids (NLs) in the two strains almost doubled, while the glycolipid (GL) contents significantly decreased. During this period, the increase in NL content was almost equivalent to the reduction in GLs (Figure 3B,C). This phenomenon suggests that GLs in CL153180 and CCMP462 served as the primary provider for the conversion to NLs under ND conditions, while phospholipids (PLs) were less important or otherwise not involved. Whether PLs or GLs were first converted to NLs under conditions of environmental stress remains controversial. Freddy and Dagmar [21] studied the effect of light and temperature on the fatty acid composition of *Pavlova lutheri* and found that PLs were the major contributor towards the turnover to triacylglycerols (TAGs, the main form of NLs). Simionato et al. [22] reported that in the absence of nitrogen, GLs in *Nannochloropsis gaditana* were more likely to be converted for the synthesis of NLs. From data obtained in this study, both CL153180 and CCMP462 preferred to convert GLs to NLs under ND conditions. Berges et al. [23] suggested that photosynthetic complexes have varying sensitivities to nutritional stress, among which photosystem II is particularly susceptible to nitrogen limitation. Meanwhile, Shin et al. [24] emphasized that ND leads to the reduced synthesis of Rubisco, and therefore the use of nicotinamide adenine dinucleotide phosphate (NADPH) in the photon reaction in the Calvin cycle is blocked, resulting in the accumulation of excess electrons in the photosynthetic electron transport chain. This causes metabolic disorders of reactive oxygen species and the formation of large amounts of oxygen-free radicals, which may inhibit photosynthesis and damage the photosynthetic membranes. GL content, the main glycerol of photosynthetic membranes, may also decrease accordingly. In addition, the continuous accumulation of oil (NLs or TAGs) helps to absorb the released fatty acids during GL degradation, as well as the excessive electrons in the electron transport chain.

**Figure 4:**
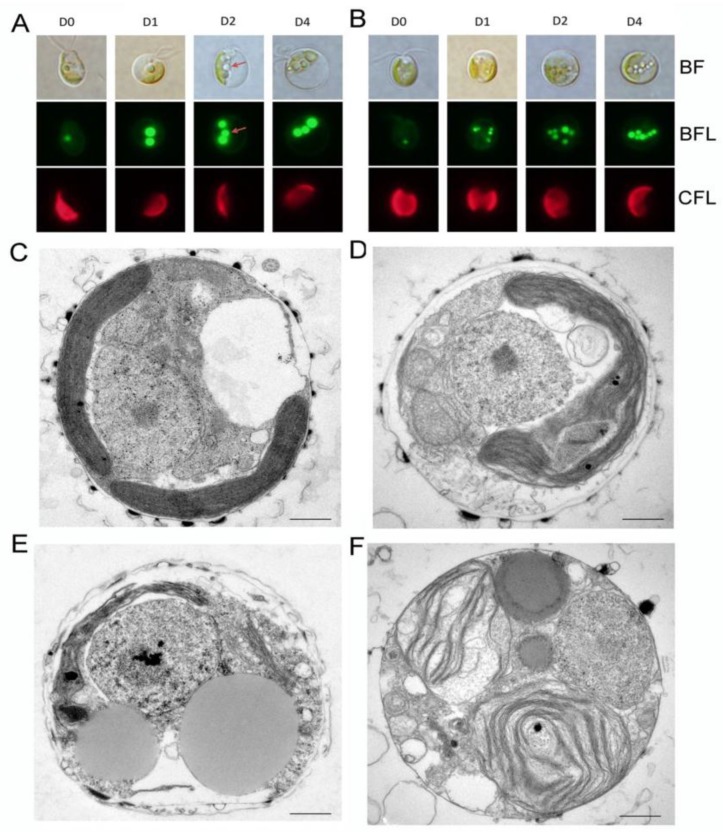
(**A**,**B**) Bodipy staining of lipid bodies; (**C**–**F**) transmission electron micrographs of CL153180 (**A**,**C**,**E**) and CCMP462 (**B**,**D**,**F**) cells under nitrogen-depleted growth conditions with continuous light illumination of 100 μmol photo m^−2^ s^−1^. D0-D4 in (**A**,**B**): nitrogen-depleted cells for 0–4 days; BF: bright field; BFL: Bodipy-stained lipid bodies fluorescence; CFL: chlorophylls autofluorescence. Bars in (**A**,**B**): 5 μm; Bars in (**C**–**F**): 0.5 μm.

In order to better examine the dynamic changes in oil in algal cells, Bodipy staining and transmission electron microscopy were performed to observe the lipid accumulation and cell structure changes under ND in CL153180 and CCMP462. As ND was prolonged, both algal cells maintained complete cell morphology with a gradual decrease in space occupied by the chloroplast. Additionally, the number and volume of oil droplets in the cells increased significantly (Figure 4A,B). Transmission electron microscopy images (Figure 4C–F) indicate the subcellular structure of CL153180 and CCMP462 on day 0 and day 4, and the changes in oil droplets in the cells can be clearly observed (Figure 4C–F). After 4 day of culture, most of the organelles were clearly discernible, but it was not clear whether the chloroplast membrane structure changed under ND conditions.

### 2.4. Distribution of DHA in Different Lipid Components

**Figure 5:**
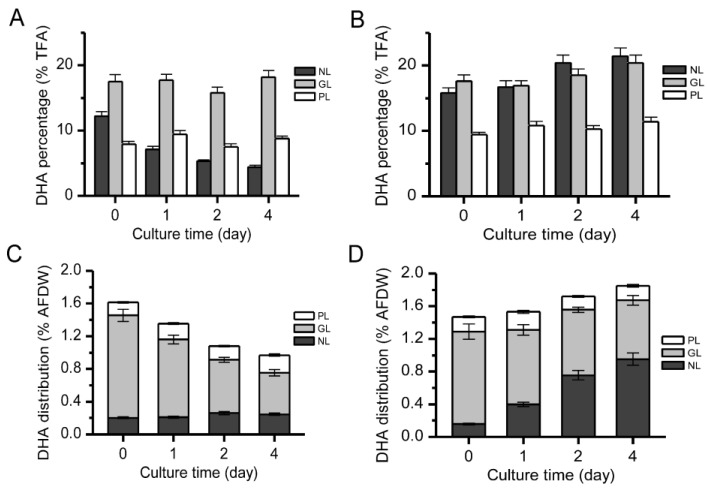
(**A**,**B**) DHA percentage; (**C**,**D**) DHA distribution in different lipid classes of CL153180 (**A**,**C**) and CCMP462 (**B**,**D**) under nitrogen-depleted growth conditions with continuous light illumination of 100 μmol photo m^−2^ s^−1^.

**Figure 6:**
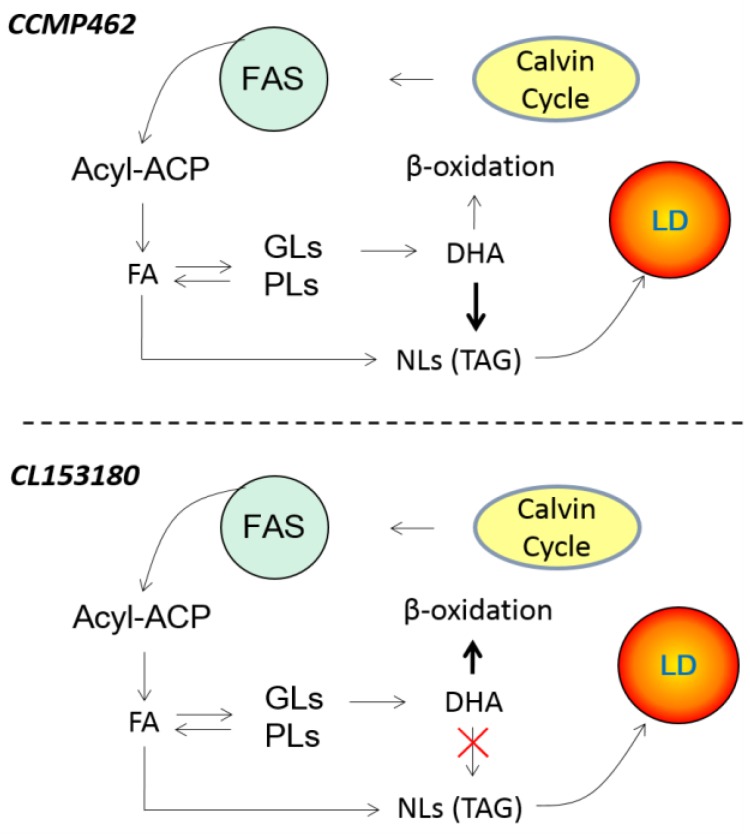
A hypothesized working model explaining the differential DHA partitioning pattern between CCMP462 and CL153180 in response to nitrogen depletion. FAS: fatty acid synthesis; LD: lipid droplet.

Turnover of lipid components can lead to the transfer of fatty acids. Therefore, the distribution of DHA in NLs, GLs, and PLs in two algal cells was evaluated. According to previous reports, DHA in *Isochrysis galbana* is mainly concentrated in GLs [13] or NLs [25], which is consistent with our findings. In CL153180, the DHA in NLs was sensitive to ND and decreased from 12.2 to 4.4% (% total fatty acids) within 4 day (Figure 5A). Conversely, in CCMP462, the DHA content in NLs increased from 15.8 to 21.4% (% total fatty acids) (Figure 5B). This difference may be attributed to the source of fatty acids in the NLs, especially TAGs. In general, the fatty acids of TAGs are derived from: (1) the de novo synthesis of fatty acids via the Kennedy pathway [26]; and (2) recycling from polar lipids [27,28]. For example, Simionato et al. [22] explored the source of fatty acids in the newly synthesized TAGs of *Nannochloropsis gaditana* under ND conditions. Using eicosapentaenoic acid (EPA, C20:5) as a signaling molecule, the change in EPA content was detected, and it was discovered that TAG synthesis mainly relied on the de novo synthesis of fatty acids, which was accompanied by the transfer of EPA from polar lipids such as monogalactosyl diglyceride (MGDG) and digalactosyl diglyceride (DGDG) to NLs. Regardless of the sources of fatty acids, acyltransferases are needed to incorporate them into NLs (mainly TAG) [29,30,31]. In the present study, both *Isochrysis* strains employ fatty acids recycled from GLs for the synthesis of NLs, but it is likely that CCMP462 utilized DHA while CL153180 did not (acyltransferases lack activity on DHA), leading to the differential DHA partitioning into NLs between the two strains (Figure 6).

The ratio of DHA to the dry weight of the algal cells in CL153180 and CCMP462 (Figure 5C,D) was also calculated. Although the DHA percentage in the NLs was decreased in CL153180, the final DHA distribution in NLs (% dry weight) remained generally unchanged due to the significant increase in the amounts of NLs. Conversely, in CCMP462, the DHA distribution in NLs (% dry weight) increased significantly, which once again confirmed the transfer of DHA from polar lipids to NLs. For both strains, the DHA distribution in GLs (% dry weight) decreased, which could be explained by the significant decrease in the amounts of GLs.

### 2.5. Effects of ND on Fatty Acid Profiles

The above results demonstrated that the NLs in these two algae were supplied with DHA from different origins under ND conditions. As a result, we further examined the changes in fatty acid profiles including DHA (Table 1). After 4 day of culture with ND, the monounsaturated fatty acids contained in CL153180 increased from 18.7 to 35.8%, significantly higher than in CCMP462. Meanwhile, the total amount of polyunsaturated fatty acids in CL153180 decreased from 43.5 to 25.8%, among which the DHA content decreased by almost half, while the percentage of DHA in CCMP462 increased by more than 30%. It is worth noting that this change is similar to stearidonic acid (C18: 4), the upstream product of DHA. In the biosynthetic pathway of DHA, Δ6-desaturase is a key enzyme that catalyzes the synthesis of C18:4^Δ6,9,12,15^ from C18:3^Δ9,12,15^. Petrie et al. [27] reported the preference of Δ6-desaturase in *Micromonas pusilla*, which showed higher activity towards C18:3^Δ9,12,15^ as a substrate than C18:2^Δ9,12^, thereby resulting in an increase in the contents of EPA and DHA (products of omega-3 pathway). Therefore, we hypothesized that the Δ6-desaturase activity in CCMP462 may be enhanced under ND conditions, which promotes the conversion efficiency from C18:3^Δ9,12,15^ to C18:4^Δ6,9,12,15^, ultimately leading to an increase in DHA content. In fact, there have been some reports demonstrating the response of Δ6-desaturases to nitrogen signals, and the mechanisms may be different [32]. Huerlimann et al. [33] selected *Isochrysis aff. galbana* as a study subject and found that the response of Δ6-desaturase was positively related to ND. It was also hypothesized by Feng et al. [34] that ND could weaken the synthesis of protein, thereby reducing the amount or activity of the enzymes that are related to the synthesis of polyunsaturated fatty acids. However, Liu et al. [35] found during their study of *Myrmecia incisa* that Δ6-desaturase was negatively correlated with ND. Whether the differences in the response of Δ6-desaturase to nitrogen deficiency in CL153180 and CCMP462 are species-specific requires further investigation.

## 3. Materials and Methods 

### 3.1. Algal Strains and Maintenance

*Isochrysis* CCMP462 (CCMP462) was obtained from Provosoli-Guillard National Centre for Culture of Marine Phytoplankton. *Isochrysis* 153180 (CL153180) was purchased from the Carolina Biological Supply Company. For maintenance, both strains were cultured in 250-mL Erlenmeyer flasks containing 100 mL F/2 medium and 25 g·L^−1^ sea salt. Cultures were kept at 22 °C with continuous illumination of a low light (20 μmol·m^−2^ s^−1^), shaken by hand once a day.

### 3.2. Algal Cultivation

Algal cultures were inoculated into column photobioreactors (PBRs, internal diameter = 3.0 cm) containing 100 mL modified F/2 medium (120 mg L^−1^ N) and grown at 22 °C, aerated with 1.5% CO_2_ enriched air (compressed air and CO_2_ are mixed at a ratio of 100:1.5), and illuminated with continuous light of 100 μmol·m^−2^ s^−1^. Sterile water was replenished every day before sampling to avoid the effect of evaporation. The cultures in late exponential growth phase were inoculated into new column PBRs with a starting cell density of 0.3 g·L^−1^. For nitrogen deficiency (ND) experiments, the same inoculation was put into the nitrogen-free F/2 medium for 4 days before being harvested. 

### 3.3. Growth Measurements

The optical density (OD) of culture was measured at 750 nm with a 1.5 cm light path cuvette in a HACH DR 2700 spectrophotometer. Culture suspension (5–10 mL) was filtered through a pre-dried Whatman GF/C filter paper (1.2 μm pore size) and washed twice with 10 mL 0.5 M NH_4_HCO_3_. Cells on the filter paper discs were dried at 100 °C in an oven until constant weight and were subsequently cooled to room temperature in a desiccator before weighing. Samples were ashed at 500 °C for 2 h in a muffle furnace to obtain ash-free dry weight (AFDW). Biomass productivity was calculated on an AFDW basis.

### 3.4. Lipid Extraction and Analysis

The extraction of total lipids was carried out in accordance with the modified method described by Bligh and Dyer [36]. Approximately 50 mg of lyophilized algae were extracted using a solvent mixture of chloroform, methanol, and water (2:1:0.8, *v*/*v*/*v*), and then vortexed for 15 min. The collected samples were centrifuged at 4000 rpm for 15 min and then the lower phase was collected into the brown tubes. The extracts were measured gravimetrically until dried to a constant weight with nitrogen gas. Dry lipid extracts were re-suspended in chloroform for immediate use or stored at −20 °C under nitrogen for later use.

Total lipid extracts were further fractionated into neutral lipids, glycolipids, and phospholipids on silica cartridges (ANPEL Scientific Co. Ltd., Shanghai, China) by sequential elution with chloroform, acetone, and methanol, as previously described [37]. The quantification of different lipid fractions was the same as that of total lipids.

### 3.5. Bodipy Staining

Approximately 1 mL of culture solution was centrifuged at 12,000 rpm for 2 min and the supernatant was discarded. Cells were stained with 1 mL Bodipy fluorescent dye (Genmed Scientific Inc., Shanghai, China) and incubated in darkness at 25 °C for 10 min. The samples were examined using a Confocal Laser Scanning Microscope (Car Zeiss, Jena, Germany) with an excitation wavelength of 490 nm and emission wavelength of 515 nm.

### 3.6. Ultrastructural Observation

Transmission electron microscopy (TEM) was applied to determine the subcellular structures of *Isochrysis*. Fresh samples were fixed with 4% paraformaldehyde and 0.5% glutaraldehyde at 4 °C for 4–12 h. The supernatant was removed and mixed with 2–5 µL egg albumen in a 15 mL-centrifuge tube. The collected algal cells were incubated with 0.5% osmic acids in 0.1 M PBS at 4 °C for 1 h. The samples were dehydrated for 10 min with ethanol and infiltrated at 25 °C with a mixture of epoxy propane and Epon812. Cells were aggregated within an aggregator (37 °C for 12 h, 45 °C for 12 h, and 60 °C for 24 h). After drying, thin sections were stained with uranyl acetate and lead for 5 min. Samples were observed under a JEOL 1230 microscope (Tokyo, Japan).

### 3.7. Fatty Acid Analysis

Fatty acid methyl esters (FAMEs) were prepared by transesterification of freeze-dried cells or individual lipids. Transesterification was conducted using 4% sulfuric acid in methanol at 85 °C for 1 h [38]. FAMEs were detected using an Agilent 7890 gas chromatography with mass spectrum (GC-MS), equipped with SP^TM^-2560 Silica Capillary Column (100 m × 0.25 mm × 0.2 um film thickness). The program was as follows: the initial temperature was maintained at 130 °C for 5 min, ramping at 4 °C min^−1^ to 220 °C for 12 min and 20 °C min^−1^ to 240 °C for 8.5 min. The injection volume was 1 µL. DHA, as well as other fatty acids, were identified by comparison with the retention time of the validated standards (Sigma-Aldrich, St. Louis, MO, USA).

### 3.8. Statistical Analyses

All experiments were determined in biological triplicate to ensure the reproducibility. Experimental results were obtained as the mean value ± SD. 

## 4. Conclusions

The two *Isochrysis* strains, CCMP462 and CL153180, showed differential patterns of DHA accumulation and partitioning into lipid classes under both nitrogen-replete and nitrogen-depleted conditions: in CCMP462, DHA increased and was enriched in NLs, while in CL153180, DHA dropped considerably without enrichment in NLs. Both strains accumulated NLs at the expense of GLs, but it is likely only CCMP462 can utilize GLs-derived DHA for the synthesis of NLs (mainly TAG), leading to the enrichment of DHA in NLs. In CL153180, the DHA released from GLs, due to its inability to be incorporated into NLs, is subject to β-oxidation for degradation. Taken together, our data provide insights into the DHA accumulation pattern and indicate that CCMP462 is superior to CL153180 for DHA production under batch culture conditions.

## Figures and Tables

**Table 1:** Fatty acid profiles of CL153180 and CCMP462 under nitrogen-depleted growth conditions.

Fatty Acids (% TFA)	CL153180				CCMP462			
	0	1	2	4	0	1	2	4
c14:0	19.4	17.4	16.7	16.7	18.9	15.9	14.1	15.5
c16:0	14.9	18.5	19.2	20.4	11.9	13.4	13.7	14.0
c16:1	4.2	2.2	2.2	2.5	5.1	4.0	3.7	3.5
c18:0	1.0	0.9	0.7	0.2	0.2	0.3	0.3	0.3
c18:1	14.5	26.5	30.9	33.3	17.4	19.9	20.1	21.9
c18:2	8.2	6.5	5.3	4.8	5.1	4.9	3.7	2.0
c18:3	9.1	6.2	4.4	3.8	9.5	7.6	5.9	4.1
c18:4	12.1	9.4	9.3	9.4	14.1	16.3	17.7	18.1
c22:0	2.4	2.6	2.3	1.0	3.3	3.0	2.9	1.6
c22:6 (DHA)	14.2	9.9	8.9	7.8	14.5	14.8	17.8	19.1
MFA ^1^	18.7	28.7	33.1	35.8	22.5	23.8	23.8	25.4
PUFA ^2^	43.5	31.9	28.0	25.8	43.2	43.7	45.1	43.2
SFA ^3^	37.7	39.4	38.9	38.4	34.3	32.5	31.0	31.4
TFA ^4^	11.9	12.9	12.5	13.2	10.9	11.2	10.3	10.1

^1^ MFA: Monounsaturated fatty acids; ^2^ PUFA: Polyunsaturated fatty acids; ^3^ SFA: Saturated fatty acids; ^4^ TFA: Total fatty acid content (% AFDW).
